# Perforated Appendicitis Masquerading as Bilateral Tubo-Ovarian Abscess

**DOI:** 10.7759/cureus.30464

**Published:** 2022-10-19

**Authors:** Humzah Iqbal

**Affiliations:** 1 Internal Medicine, University of California San Francisco, Fresno, USA

**Keywords:** tubo-ovarian abscess, pelvic inflammatory disease, appendiceal perforation, acute diarrhea, appendicitis

## Abstract

Acute appendicitis is a highly common cause of abdominal pain that presents with nausea and vomiting, characteristic physical exam findings, and imaging evidence of appendiceal inflammation. In the absence of these signs, diagnosis can be difficult. This case report demonstrates an uncommon presentation of appendicitis that led to a delay in diagnosis and aims to discuss the ways in which similar misdiagnoses can be avoided for clinicians in the future.

## Introduction

Acute appendicitis is a highly prevalent condition that serves as one of the most common causes of acute abdominal pain with a lifetime risk of 8.6% in males and 6.7% in females [1]. It most often presents with peri-umbilical pain, right lower quadrant pain, abdominal rigidity, and fever. A tubo-ovarian abscess (TOA) is a more rare condition that is often a late-stage presentation of pelvic inflammatory disease (PID). Patients with TOA present with acute onset lower abdominal pain, fever, and evidence of pelvic abscess on imaging. Diarrhoea is not a common presenting symptom for either condition, however, it is more often seen as a result of gastroenteritis. Physical exam findings that are considered to be specific in the diagnosis of appendicitis include Rovsing’s sign, psoas sign, and obturator sign. This case is a unique presentation of acute appendicitis complicated by perforation which had none of the classic physical exam findings and was diagnosed as bilateral TOA with possible concurrent gastroenteritis. The patient was not accurately diagnosed until almost a week later, which prevented immediate operative management of her condition. She was eventually treated non-operatively. Treatment of complicated appendicitis is achieved by either operative management with appendectomy or conservative management with antibiotics and percutaneous drainage when warranted [2]. A randomized control trial by Mentula et al. found that immediate laparoscopic appendectomy in perforated appendicitis with abscess (median symptom duration of seven days) resulted in fewer readmissions and fewer additional interventions [3]. Additionally, a study by Elkbuli et al. showed that non-operative management of complicated appendicitis is associated with increased rates of treatment failure and morbidity [4]. However, some studies have suggested that conservative management is associated with a decrease in morbidity [2,5]. This case report aims to highlight an atypical presentation of appendicitis that was successfully treated non-operatively. Clinicians should be aware of the potential for unusual presentations in order to provide patients with the most appropriate care without unnecessary delay. This is particularly crucial in conditions where a delay in care can lead to a significant increase in morbidity and/or mortality.

## Case presentation

A 21-year-old female patient with no significant past medical history presented to the Emergency Department with acute onset abdominal pain for the past three days. She reported having multiple sexual partners and sexual intercourse with a new partner three days ago and immediately afterward developed crampy diffuse abdominal pain. The patient also reported the onset of non-bloody watery diarrhea associated with bowel incontinence the day before the presentation. She denied any sick contacts, recent travel, fever, chills, nausea, vomiting, and dysuria. She denied any vaginal discharge, malodor, and previous history of sexually transmitted infection (STI).

**Figure 1:**
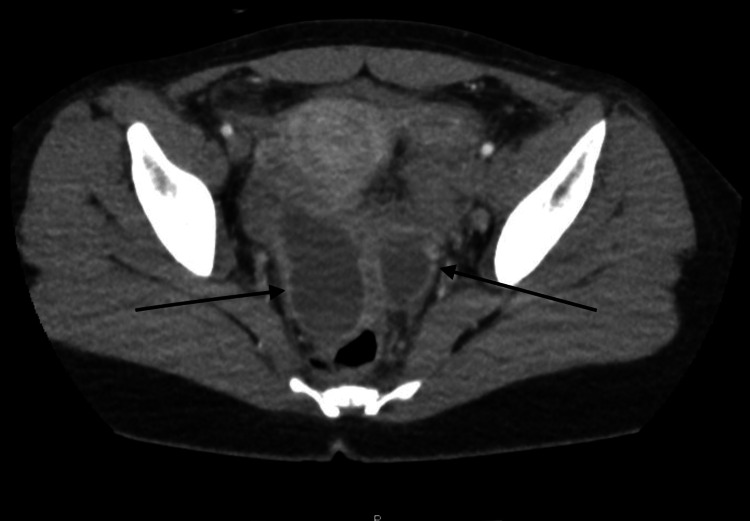
CT abdomen/pelvis (axial plane) The image shows bilateral fluid collections (arrows) suggestive of bilateral tubo-ovarian abscess.

**Figure 2:**
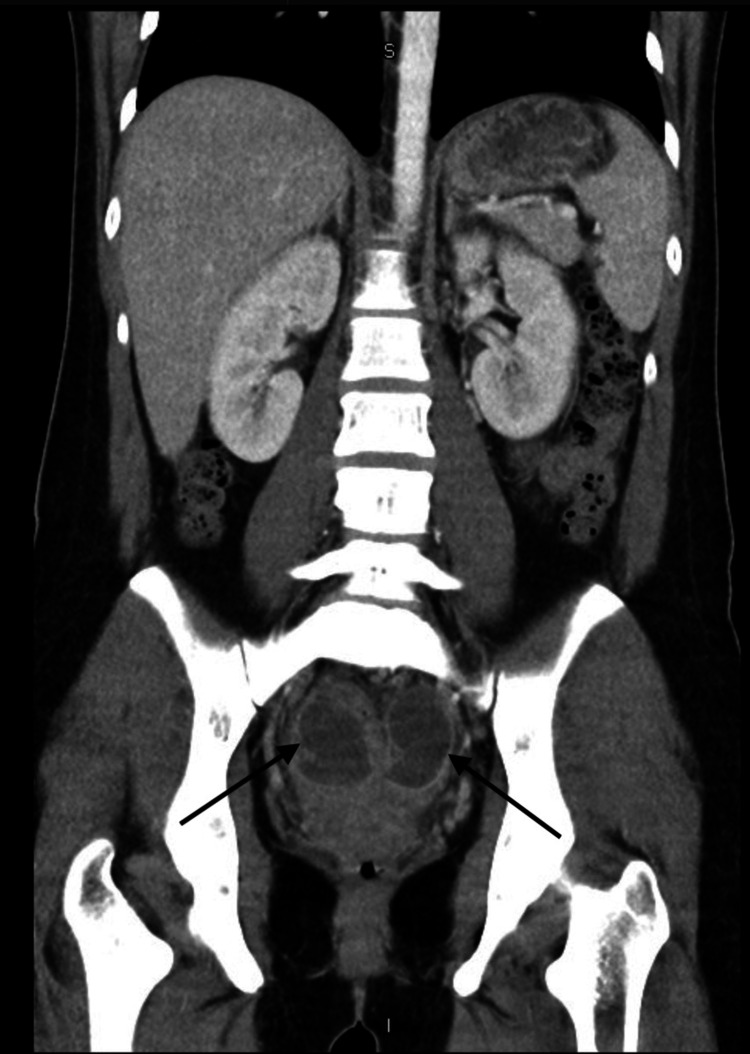
CT abdomen/pelvis (coronal plane) The image is demonstrating bilateral fluid collections (arrows) concerning tubo-ovarian abscesses.

**Figure 3:**
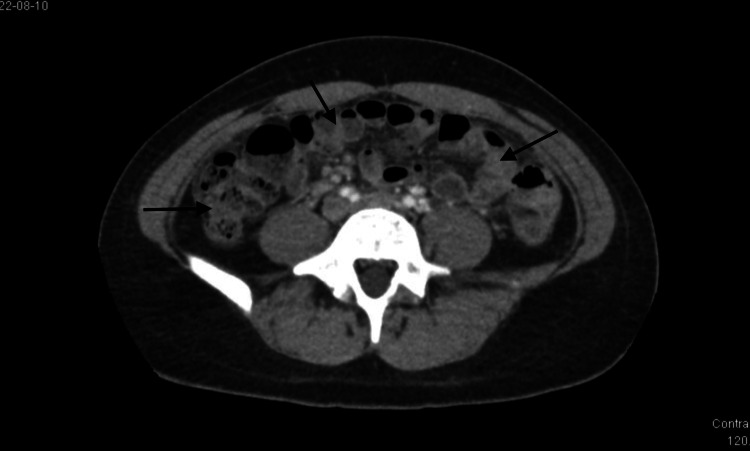
CT abdomen/pelvis (axial plane) The image is demonstrating evidence of intestinal inflammation (arrows) concerning enteritis.

The patient’s vital signs on presentation revealed a temperature of 37.9°C and a heart rate of 115 beats per minute. On physical examination, the patient had tenderness to palpation in the lower quadrants of the abdomen bilaterally, with greater tenderness in the left lower quadrant. She denied peri-umbilical pain. Bowel sounds were present and the abdomen was tympanic to percussion. Physical exam was negative for Rovsing’s sign, psoas sign, and obturator sign. A complete blood count was obtained which was notable for leukocytosis of 19.3 x 10^9^/L. Computed tomography (CT) scan of the abdomen/pelvis was obtained which showed tubular complex fluid collections in bilateral adnexal regions, suggestive of bilateral TOA (Figures 1-2). The CT imaging also showed multiple distended loops of small bowel with thickened mucosa (Figure 3). The appendix was not visualized. 

Treatment was initiated for TOA with metronidazole and doxycycline, and then ceftriaxone was started after 24 hours for possible gastroenteritis. Stool studies were negative, and the patient began to show clinical improvement with antibiotics. Leukocytosis, pain, fever, and incontinence all showed improvement after 48 hours. After five days of this regimen with progressive improvement, the patient was planned to be discharged with oral antibiotics to continue at home. However, on the day of discharge, her complete blood count was significant for normal white blood cell count with a band cell count of 19%. This was concerning for ongoing infection, as patients with bandemia without leukocytosis are at higher risk for infection and/or death [6]. Therefore, a repeat CT abdomen/pelvis was obtained which showed a re-demonstration of the fluid collections with findings indicative of appendicitis with a ruptured appendiceal tip, which was established as the patient’s true diagnosis. Interventional radiology was consulted and CT-guided drainage of the abscess was performed. Approximately 30 mL of purulent fluid was drained and a drainage catheter was placed.

**Figure 4:**
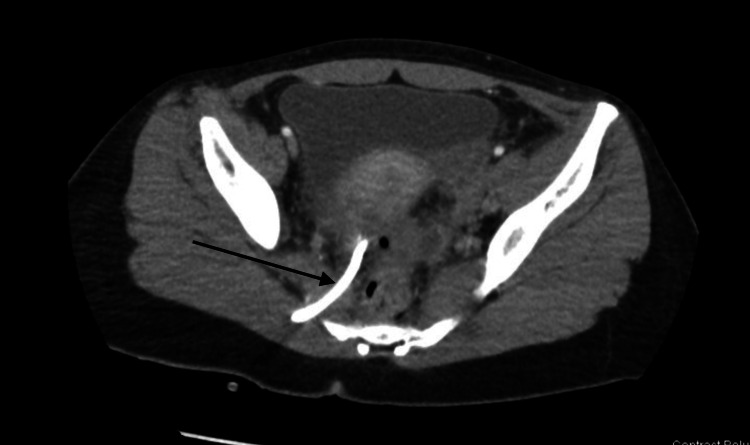
CT abdomen/pelvis The image is showing resolution of the fluid collection with the presence of a drainage catheter (arrow).

The patient felt an immediate improvement in her symptoms following the CT-guided drainage of her pelvic abscess. Repeat CT abdomen/pelvis showed resolution of the dominant collection in the right pelvis (Figure 4). The patient’s bandemia resolved and her diarrhea and pain continued to improve. She was discharged with the drainage catheter in place, which had minimal output, and oral amoxicillin-clavulanic acid to complete a 14-day course of antibiotic treatment. She was given instructions to return in five days for the removal of the drain, which was removed without issue.

## Discussion

This case demonstrates a common condition with a unique presentation, leading to a delay in diagnosis. Based on her acute onset abdominal pain in the lower quadrants, history of multiple sexual partners including a new sexual partner recently, and the presence of bilateral pelvic abscesses on imaging “suggestive of bilateral tubo-ovarian abscess,” the true diagnosis was masked. Atypical presentations of appendicitis are not uncommon, and it is crucial for clinicians to be aware of this when formulating differential diagnoses [7]. Acute appendicitis most often presents as acute abdominal pain in the peri-umbilical region and/or right lower quadrant. One of the complications of acute appendicitis is perforation of the appendix, which can lead to constipation and severe nausea and vomiting, along with worsening abdominal pain [7]. The patient presented with diarrhea as one of her chief complaints and did not have any of the classic physical exam findings, making appendicitis unlikely. A prospective study by Santillanes et al. found the likelihood ratios of Rovsing’s sign, psoas sign, and obturator sign for appendicitis to be 3.94, 3.15, and 3.52 respectively [8].

The patient’s age group and sexual history do put her at increased risk for pelvic inflammatory disease (PID) and therefore TOA, which was corroborated by CT findings [9]. In addition, TOA can lead to bowel wall thickening on imaging due to secondary inflammation, as seen in this case [10]. However, it is highly important to consider the pre-test probability of a differential when assessing a patient. This patient had no history of PID or STI, and TOA is not as common of a condition overall which occurs in about 17-20% of patients with PID, which has a worldwide incidence of about 1 million per year [9]. In comparison, appendicitis has a worldwide incidence of 17.7 million cases per year [11]. In young patients with acute abdominal pain, appendicitis should ideally be considered as a possibility. Nevertheless, the patient's atypical presentation made it a challenging diagnosis.

Imaging is a highly useful adjunct during the stage of diagnostic reasoning, however, it may not always provide the correct diagnosis and should not be treated as a substitute for clinical reasoning. In a case report presented by Taylor et al., a 47-year-old female developed a TOA that was misdiagnosed as an appendiceal abscess based on CT findings, leading to a delay in care [12]. Similarly, the patient in this case report had treatment for TOA and gastroenteritis which delayed her true diagnosis for almost one week. Patients with acute appendicitis that is left untreated for greater than 72 hours are at the greatest risk for perforation [13]. This time course fits with the patient’s history as she developed unbearable abdominal pain about three days after the onset of symptoms, likely corresponding to appendiceal rupture that prompted her presentation to the Emergency Department. If suspicion of appendicitis had been higher, this course may have been recognized sooner and the pelvic abscesses may have been correctly attributed to being a sequela of appendiceal perforation. It is crucial for clinicians to be aware of atypical presentations of disease states in order to initiate timely and appropriate management.

Studies have shown mixed results in regard to operative versus non-operative treatment of complicated appendicitis. However, a systematic review and meta-analysis of 17 studies by Simillis et al. demonstrated that non-operative management of complicated appendicitis led to a lower reoperation rate and decrease in complications while maintaining a similar length of hospital stay as compared to immediate surgical intervention [5]. Conservative management includes antibiotics and percutaneous drainage, as seen in this case, and has been increasing in usage amongst clinicians [14]. A study by Maxfield et al. found that patients who present with tachycardia and generalized abdominal pain have an increased risk of failing the non-operative management of complicated appendicitis [15]. However, this was not observed in this patient who was successfully treated with conservative management despite presenting with these symptoms.

## Conclusions

Acute appendicitis in the absence of classical features can be a challenging diagnosis that mimics other conditions. Clinicians should be aware of these unusual presentations and assess the patient's overall clinical picture rather than solely depending on imaging findings, in order to avoid misdiagnoses and delays in care. Once the diagnosis of complicated appendicitis has been made, conservative treatment with percutaneous drainage and antibiotics without operative management can be initiated.
